# Odd-Chain Fatty Acids-Enriched Algal Oil Improves Locomotor Function and Modulates Metabolic Pathways in *Caenorhabditis elegans* Model of Alzheimer’s Disease

**DOI:** 10.3390/molecules31101734

**Published:** 2026-05-19

**Authors:** Qin Mu, Yiwei Ma, Tao Zhang, Fang Cong, Jun Jin, Qingzhe Jin, Xingguo Wang

**Affiliations:** 1 State Key Laboratory of Food Science and Resources, School of Food Science and Technology, Jiangnan University, Wuxi 214122, China; 6230112063@stu.jiangnan.edu.cn (Q.M.); jqzwuxi@163.com (Q.J.); xingguow@jiangnan.edu.cn (X.W.); 2 Wilmar (Shanghai) Biotechnology Research & Development Center Co., Ltd., Shanghai 200137, China; mayiwei@cn.wilmar-intl.com; 3 College of Food Science and Technology, Huazhong Agricultural University, Wuhan 430070, China; 4 Food Laboratory of Zhongyuan, Luohe 462300, China

**Keywords:** odd-chain fatty acids, algal oil, Alzheimer’s disease, untargeted metabolomics

## Abstract

Alzheimer’s disease (AD) is a common age-related neurodegenerative disorder with extremely low drug development success rates, making nutritional intervention a promising strategy. Cerebral energy metabolism dysfunction is a core pathological feature of AD. Odd-chain fatty acids (OCFAs) can generate propionyl-CoA via β-oxidation to replenish the impaired tricarboxylic acid (TCA) cycle. This study characterized the lipid composition of OCFAs-enriched algal oil by UPC^2^-Q-TOF-MS, evaluated its neuroprotective effects on *Caenorhabditis elegans* (*C. elegans*) models with AD, Parkinson’s disease (PD), and Huntington’s disease (HD), and explored the metabolic mechanism of its key component pentadecanoic acid (C15:0) using untargeted metabolomics. Results showed that triglycerides (TAGs) represented the predominant lipid class, accounting for 97.3% of the total lipid content in the algal oil. Among all the identified TAG molecular species, TAGs containing C15:0/C17:0 accounted for more than 90%. OCFAs-enriched algal oil exhibited disease-selective neuroprotection. It significantly improved locomotor function in AD nematodes, moderately ameliorated PD-related deficits, whereas showed no efficacy in HD nematodes. Metabolomics revealed that C15:0 produced propionyl-CoA to rescue TCA cycle dysfunction and energy deficits, upregulated membrane phospholipids to repair membrane integrity, and reduced abnormal metabolites to restore metabolic homeostasis. KEGG analysis confirmed that C15:0 globally regulated core metabolic pathways including amino acid, cofactor, nucleotide, and carbon metabolism. OCFAs-enriched algal oil exerted selective anti-AD effects by repairing energy metabolism, remodeling membrane phospholipids, and restoring metabolic homeostasis, providing a novel nutritional candidate for AD intervention.

## 1. Introduction

Neurodegenerative diseases are a group of progressive neurological disorders closely related to aging, with Alzheimer’s disease (AD), Parkinson’s disease (PD), and Huntington’s disease (HD) as the main representatives. They have become a major public health problem threatening the health of the elderly. These diseases share the common feature of the progressive loss and dysfunction of specific neuronal populations in the central nervous system, leading to irreversible decline in motor, cognitive, and autonomic nerve functions. However, the exact pathogenesis of AD, PD, and HD remains incompletely understood, and the three diseases have significant differences in pathological features and clinical manifestations. Nevertheless, they share several key pathophysiological processes, such as mitochondrial dysfunction, abnormal energy metabolism, oxidative stress, neuroinflammation, and protein homeostasis imbalance [1]. Notably, over the past two decades, the failure rate of drug development pipelines targeting these core pathological links is high, and existing clinical drugs can only partially alleviate symptoms without preventing or reversing disease progression. Moreover, long-term use often comes with severe side effects [2,3]. Therefore, in the face of the current reality of a lack of effective drug treatment options, non-pharmaceutical nutritional intervention strategies aimed at preventing or delaying disease onset have become an urgent and promising research direction in the field of neurodegenerative disease prevention and control, due to their safety, accessibility, and long-term applicability.

Alzheimer’s disease (AD) is an age-related neurodegenerative disorder characterized by insidious onset and prolonged latency, and it constitutes one of the primary etiologies of dementia [4]. Currently, AD has become the fifth leading cause of death worldwide. Data from the Global Burden of Disease Study 2016 (GBD 2016) shows that in 2016, the global death toll from dementia reached nearly 2 million, with AD-related deaths dominating [5]. Due to the fact that the exact pathogenesis of AD has not been fully uncovered, there has always been a lack of radical measures in clinical practice that can effectively prevent or reverse the disease process. Currently, the failure rate of drug development for AD is high, and many clinical trials over the past two decades have produced negative results [2,6]. The drugs currently approved for clinical use can only alleviate AD symptoms to a certain extent, failing to reverse or cure the disease. Furthermore, they may be associated with severe adverse effects, including gastrointestinal complications, dizziness, insomnia, muscle spasms, and abnormal heart rate, among other notable reactions [3]. Therefore, in the absence of effective drug treatment measures, early non-pharmaceutical nutritional intervention aimed at preventing or delaying the onset of the disease is of crucial importance.

Over the past few decades, emerging multidisciplinary metabolism research has highlighted that metabolic homeostasis is fundamental to cellular function and organismal health, and its dysregulation acts as a unifying pathogenic driver for both metabolic disorders and neurodegenerative diseases [7]. Clinical studies have consistently demonstrated a close association between cerebral energy insufficiency and cognitive impairment in patients with AD [8,9,10,11]. The brain, despite comprising just over 2% of human body weight, consumes approximately 20% of the total energy supply [12]. To sustain this demand, it requires a constant supply of energy in the form of ATP. Most ATP is generated from glucose via mitochondrial oxidative phosphorylation, which occurs sequentially downstream of aerobic glycolysis in the cytoplasm [13,14]. However, in neurodegenerative diseases associated with aging, brain glucose metabolism deteriorates in a progressive, region-specific, and disease-specific manner [15]. Such decline is particularly pronounced in AD, often manifesting even before clinical symptoms emerge. Impaired brain energy metabolism plays a significant role in both the development and progression of AD [16]. Persistent deficits in brain energy interact with neuropathological processes, creating a self-reinforcing cycle that exacerbates energy depletion and dysfunction. Notably, neurons mainly rely on mitochondrial glucose oxidation as the primary source of ATP [17]. Thus, mitigating energy deficiency holds considerable promise for the therapeutic intervention of AD [18,19].

Notably, a range of nutritional supplements and dietary components have been demonstrated to exert beneficial effects on the prevention and management of AD [20]. For example, docosahexaenoic acid (DHA) is an omega-3 long-chain polyunsaturated fatty acid highly enriched in the gray matter of the brain. As a major structural component of neuronal membranes in the form of phospholipids, it plays critical roles in maintaining membrane fluidity and stability, regulating gene expression, facilitating neurotransmitter release, supporting transmembrane receptor function, and mediating signal transduction [21,22]. As a new type of functional fatty acid, odd-chain fatty acids (OCFAs) have long been regarded as biomarkers of dairy fat intake in adipose tissue, as their concentrations in plasma and red blood cells are positively correlated with dairy fat intake [23,24]. Recent cohort and case-control studies have revealed inverse correlations between circulating OCFAs levels and the incidences of type 2 diabetes, cardiovascular disease, metabolic dysfunction-associated steatohepatitis, heart diseases, and specific cancer subtypes [25,26,27,28,29,30,31,32]. The observed correlation between circulating C15:0 levels and reduced incidence of multiple pathologies suggests that dietary supplementation with OCFAs may exert protective effects against these conditions [33,34,35,36]. Unlike even-chain fatty acids, OCFAs can be metabolized through β-oxidation to generate acetyl-CoA and propionyl-CoA, the latter of which can be converted into succinyl-CoA and directly enter the TCA cycle for energy production [37]. Succinyl-CoA is an important intermediate metabolite in the TCA cycle for the synthesis of succinate, and succinate deficiency leads to the blockage of the TCA cycle, especially in AD. Studies have shown that the additional supplementation of succinyl-CoA can bypass the glucose metabolic pathway and intentionally drive the impaired mitochondrial tricarboxylic acid cycle [38]. Thus, in the present study, an algal oil enriched in OCFAs was employed, and the AD model of *Caenorhabditis elegans* (*C. elegans*) was utilized to investigate the efficacy and underlying mechanisms of OCFAs in the context of AD.

## 2. Results and Discussion

### 2.1. Analysis of Glyceride Composition of the Algal Oil

Triglycerides (TAGs) were the predominant lipid class in OCFAs-enriched algal oil, accounting for 97.3 ± 0.6% of the total lipid mass. By contrast, the levels of free fatty acids (FFAs) and diacylglycerides (DAGs) were low, at 0.9 ± 0.1% and 1.2 ± 0.2%, respectively. Other components, such as monoglycerides (MAGs), accounted for only 0.3 ± 0.1%, confirming that TAGs constitute the major acylglyceride species in this algal oil (Table 1).

### 2.2. Qualitative Determination of TAGs of the Algal Oil

**Figure 1:**
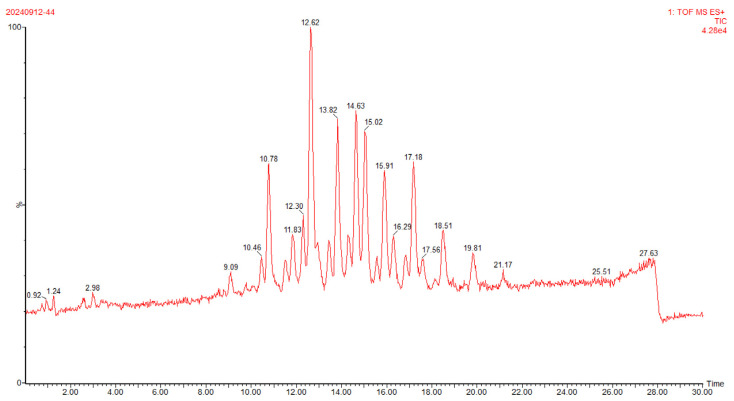
The total ion chromatogram of triacylglycerides from algal oil enriched in OCFAs by UPC^2^-Q-TOF-MS.

**Figure 2:**
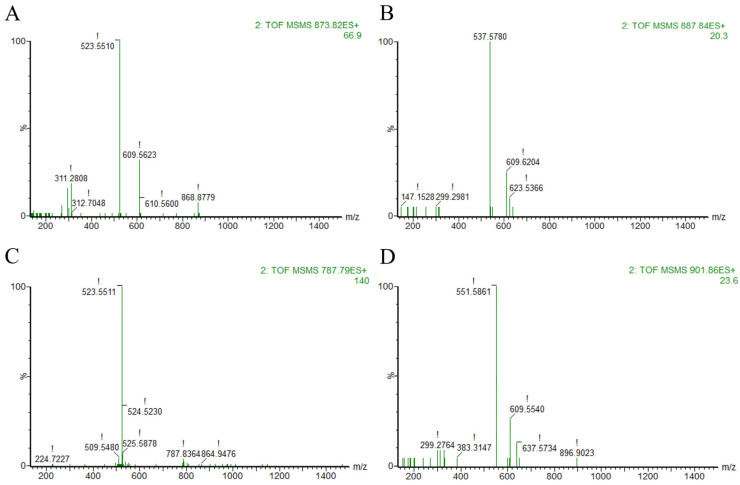
MS/MS spectrum at the retention time of DHA-C15:0-C15:0 at 12.62 min (**A**), DHA-C16:0-C15:0 at 13.82 min (**B**), C15:0-C15:0-C15:0 at 14.63 min (**C**) and DHA-C17:0-C15:0 at 15.02 min (**D**).

To further characterize the structure and composition of TAGs in the algal oil which is rich in OCFAs, ultra-performance convergence chromatography coupled with quadrupole time-of-flight mass spectrometry (UPC^2^-Q-TOF-MS) was employed for compositional analysis. The corresponding total ion chromatogram (TIC) and high-energy state mass spectra (MS/MS) are presented in Figure 1 and Figure 2.

As shown in Figure 1, chromatographic peaks achieved baseline separation within 30 min, with excellent resolution between adjacent peaks. Mass spectrometry analysis employed the MS^E^ acquisition mode, which enabled the capture of accurate mass data for precursor ions and their corresponding product ions via programmed alternation of low- and high-energy collision voltages during a single full-scan injection. In ESI positive ion mode, the signal intensity of [M + Na]^+^ was substantially higher than that of [M + NH_4_] ^+^, thus [M + Na]^+^ was selected as the precursor ion for tandem mass spectrometry (MS/MS) analysis. The identification workflow for TAG molecular species proceeded as follows: first, the value of the molecular ion peak [M + Na]^+^ in the low-energy total ion chromatogram of MSE in ESI+ mode was observed to preliminarily determine the relative molecular mass of the molecular ion peak. Then, the relative molecular mass of the fatty acid was calculated based on the value of the corresponding fragment ion in the high-energy state, and finally the type of TAG was determined.

As exemplified by the chromatographic peak eluting at 12.62 min (Figure 2A), the [M + Na] ^+^ quasimolecular ion was detected at *m*/*z* 873.9129 in the low-energy MS^E^ spectrum, and the two fragment ion peaks under high energy state are *m*/*z* 523.551 and 609.5623 respectively. Mass difference calculations yielded fatty acyl residue masses of 328.36 and 242.35 Da, corresponding precisely to docosahexaenoic acid (DHA; theoretical mass 328.23 Da) and pentadecanoic acid (C15:0, theoretical mass 242.23 Da). This unambiguously identifies the TAG as a DHA-C15:0-C15:0 molecular species. Analogous analysis of peaks at 13.82 min, 14.63 min, and 15.02 min assigned the molecular species as DHA-C16:0-C15:0, C15:0-C15:0-C15:0, and DHA-C17:0-C15:0, respectively.

### 2.3. Quantification of TAGs of the Algal Oil Enriched in OCFAs

The TAG molecular species profile of the OCFAs-enriched algal oil was determined by UPC^2^-Q-TOF-MS and is presented in Table 2.

According to the table, a total of 25 main TAG species were identified in the OCFAs-enriched algal oil. Structural elucidation was primarily performed based on retention time, [M + Na]^+^ quasi-molecular ion peaks, equivalent carbon number (ECN), and secondary fragment ion data. In terms of compositional characteristics, the TAGs in C15:0 algal oil exhibited a concentrated carbon chain length distribution. The most abundant species was DHA-C15:0-C15:0 (19.48%), followed by DHA-C17:0-C15:0 (11.20%), DHA-C16:0-C15:0 (10.04%), and DHA-DHA-C15:0 (8.17%). Additionally, a relatively high content of fully saturated triglyceride C15:0-C15:0-C15:0 (8.60%) was detected, covering a wide range of compositions from low-carbon to long-chain polyunsaturated fatty acids.

Notably, TAG species with identical ECN values (e.g., those eluting at retention times of 13.50 min and 15.91 min) were still well resolved under the applied chromatographic conditions, demonstrating the high resolving power of the UPC^2^-Q-TOF-MS method for complex TAG mixtures. Overall, TAGs co-esterified with OCFAs dominating the C15:0 algal oil, and the cumulative relative content of TAGs containing C15:0 and C17:0 exceeded 90%, which provided a well-defined material basis for subsequent investigations into its biological activities.

### 2.4. Effects of OCFAs-Enriched Algal Oil on Locomotory Capacity of C. elegans Model with Neurodegenerative Disease

Motor dysfunction is a core phenotypic hallmark of neurodegenerative diseases, directly reflecting the extent of neuronal structural compromise and functional impairment. In *C. elegans*, body bend frequency is widely established as a quantitative, behaviorally relevant metric of locomotor performance. It provides a sensitive and interpretable readout of neural integrity and the efficacy of exogenous bioactive interventions, and has been extensively adopted in mechanistic studies of neurodegeneration [39]. To comparatively assess the therapeutic potential of lipid-based interventions, three transgenic *C. elegans* models were employed: Alzheimer’s disease (strain GMC101), Parkinson’s disease (strain NL5901), and Huntington’s disease (strain AM141). The effects of OCFAs-enriched algal oil versus DHA-enriched algal oil on locomotory performance were evaluated.

**Figure 3:**
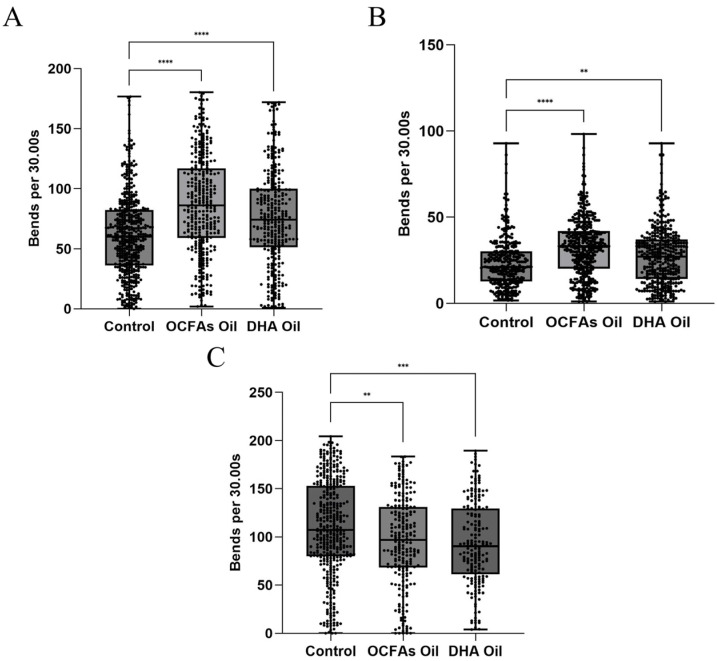
Effects of OCFAs-enriched algal oil and DHA-enriched algal oil on bend frequency of AD *C. elegans* (**A**); PD *C. elegans* (**B**); HD *C. elegans* (**C**). ** (*p* < 0.01), *** (*p* < 0.001), **** (*p* < 0.0001).

For the AD model (Figure 3A), both OCFAs-enriched algal oil and DHA-enriched algal oil interventions significantly elevated the body bend frequency of AD nematodes relative to the control group (*p* < 0.0001). However, a statistically significant difference was observed between the two interventions: the OCFAs-enriched algal oil group exhibited a 31.6–38.2% increase in body bend frequency compared to the control (with high consistency across parallel replicates), whereas the DHA-enriched algal oil group showed a 20.3–25.7% increase (*p* < 0.0001). Notably, the ameliorative effect of OCFAs-enriched algal oil was significantly superior to that of DHA-enriched algal oil, indicating that OCFAs exert a significant mitigating effect on the motor deficits of AD model nematodes.

For the PD model (Figure 3B), both OCFAs-enriched algal oil and DHA-enriched algal oil interventions significantly elevated the body bend frequency of PD nematodes relative to the control group. A statistically significant difference was observed between the two interventions: the OCFAs-enriched algal oil group induced a 28.5–35.1% increase in body bend frequency in PD nematodes compared to the control (*p* < 0.0001), whereas the DHA-enriched algal oil group elicited a 17.6–22.3% increase (*p* < 0.01). Notably, the ameliorative effect of OCFAs-enriched algal oil was significantly superior to that of DHA-enriched algal oil, indicating that OCFAs also possess neuroprotective potential in the PD model.

For the HD model (Figure 3C), both the OCFAs-enriched algal oil and DHA-enriched algal oil interventions significantly reduced the body bend frequency of HD nematodes relative to the control group (*p* < 0.01). Data from parallel replicates exhibited minimal variability, indicating that neither of the two oils improved the motor dysfunction of the HD nematodes and even had an inhibitory effect. Furthermore, these findings suggested that OCFAs lacked neuroprotective potential in the HD model.

In summary, OCFAs-enriched algal oil exhibited distinct disease model selectivity in ameliorating locomotor dysfunction across three of the neurodegenerative disease model nematodes. It had the most significant effect on AD nematodes, followed by PD models, and showed no significant efficacy in HD models. These findings suggested that the neuroprotective activity of OCFAs-enriched algal oil might stem from the specific modulation of disease-associated pathological pathways, rather than a broad-spectrum neuroprotective effect. Subsequent studies focused on C15:0, the signature OCFA in the algal oil, to dissect its intervention mechanism in AD at the transcriptional level.

### 2.5. Effects of OCFAs-Enriched Algal Oil on β-Amyloid Deposition in AD Model C. elegans

**Figure 4:**
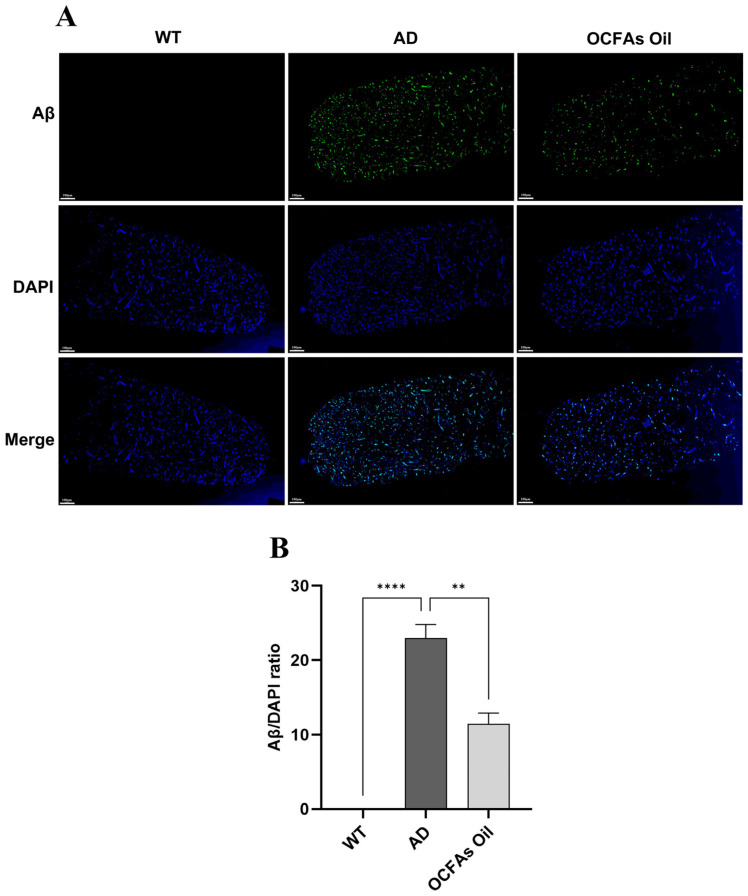
(**A**) Immunofluorescence images of the heads of wild-type (WT) and AD *C. elegans* (Scale bar = 100 μm); (**B**) Quantification of relative Aβ fluorescence in *C. elegans.* ** (*p* < 0.01), **** (*p* < 0.0001).

To clarify the regulatory effect of OCFA-rich algal oil on the core pathological feature of Aβ deposition in AD nematodes, the heads of the nematodes were stained with anti-Aβ1-42 antibody for immunofluorescence, and images were collected by confocal microscopy and analyzed for relative fluorescence intensity quantitatively. The results are shown in Figure 4.

To validate the AD nematode model, we first assessed Aβ deposition in the head region of the healthy control strain CL2122 (WT; Wild Type) and the AD model group. No distinct Aβ-positive fluorescent signals were detected in the heads of WT nematodes. In contrast, the AD model nematodes exhibited dense Aβ immunofluorescent plaques in the head, with the relative fluorescence intensity being extremely significantly higher than that of the WT group (*p* < 0.0001), confirming the successful establishment of the AD nematode model. For the intervention group treated with OCFA, the intensity of Aβ immunofluorescent signals in the nematode heads was significantly reduced (*p* < 0.01). These results indicated that OCFAs could effectively suppress the abnormal aggregation of Aβ in the brains of AD nematodes and mitigate AD-associated pathological alterations.

### 2.6. Untargeted Metabolomic Analysis of Significantly Altered Metabolites Following OCFA Intervention

#### 2.6.1. Metabolites Multivariate Analysis

**Figure 5:**
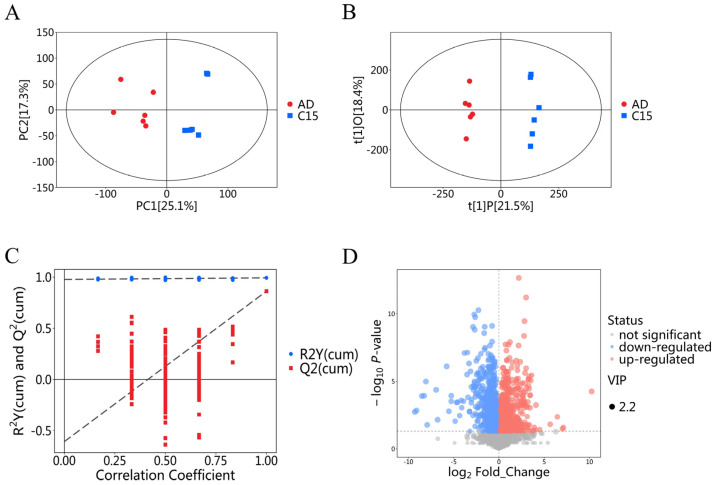
Multivariate analysis of metabolites between the AD group and C15:0 intervention group nematodes. (**A**) Score plot of PCA, (**B**) Score plot of OPLS-DA, (**C**) OPLS-DA model validation, and (**D**) Volcano plot of AD vs. C15:0 groups. Each dot represents a metabolite: Red = upregulated metabolites; blue = downregulated metabolites; and gray = metabolites with nonsignificant differences.

Metabolomics analysis was performed to characterize the metabolomic differences between AD model nematodes and C15:0-treated nematodes. PCA of the metabolomic dataset showed that biological replicates within each group exhibited favorable clustering with acceptable reproducibility (Figure 5A). OPLS-DA demonstrated partial separation between the two groups, indicating distinct metabolic profiles in AD model nematodes versus C15:0-treated nematodes (Figure 5B). The original OPLS-DA model showed excellent goodness-of-fit and predictive ability, with R^2^Y = 0.993 and Q^2^ = 0.862, indicating a clear separation of metabolic profiles between the C15:0 intervention group and the control group. To validate the model and exclude overfitting, a permutation test with 200 iterations was performed. The validation results showed that all permuted R^2^ and Q^2^ values were lower than the original values, and the intercept of Q^2^ was −0.61 (Figure 5C). These results collectively confirmed the reliability and absence of the overfitting of the OPLS-DA model.

#### 2.6.2. Differential Metabolites Analysis

The volcano plot visualized 1385 metabolites that were differentially abundant between AD model nematodes and C15:0-treated nematodes (Figure 5D), with 791 metabolites upregulated and 594 downregulated in the C15:0-treated group relative to the AD model group. Each data point corresponded to an individual metabolite: red dots denoted upregulated metabolites, blue dots denoted downregulated metabolites, and gray dots represented metabolites with non-significant changes. The top 20 most impactful differential metabolites were identified and provisionally proposed as potential biomarkers of C15:0 intervention for subsequent validation (Table 3).

As indicated in Table 3, propionyl-CoA levels were significantly elevated following C15:0 intervention, directly confirming that C15:0 undergoes β-oxidation to specifically generate propionyl-CoA. This metabolite directly replenishes the tricarboxylic acid (TCA) cycle impaired in AD, providing direct metabolic evidence that C15:0 rescues brain energy deficits. The significant upregulation of 2-hydroxybutyrate demonstrated that lipid metabolism-mediated energy supply pathways were reactivated after C15:0 intervention. Lysophosphatidylglycerol (LPG 4:0), phosphatidylcholine (PC 39:6; PC39:7), and phosphatidylinositol (PI 2:0_7:0) were all significantly upregulated, suggesting that C15:0 promoted the synthesis and remodeling of membrane phospholipids, repaired membrane integrity, and maintained membrane fluidity and neural signal transduction. Monoglyceride 15:1 (MG 15:1) is an intermediate of lipolysis; LDGTS 10:0 and MGDG O-13:1_2:0 are plant-derived abnormal glycolipids; chromene derivatives and adenine derivatives are aberrant metabolites of nucleic acids and heterocyclic compounds. All these abnormal metabolites exhibited a downward trend, indicating that C15:0 restored global metabolic homeostasis and reduced the production of aberrant metabolites.

#### 2.6.3. Metabolic Pathway Analysis

**Figure 6:**
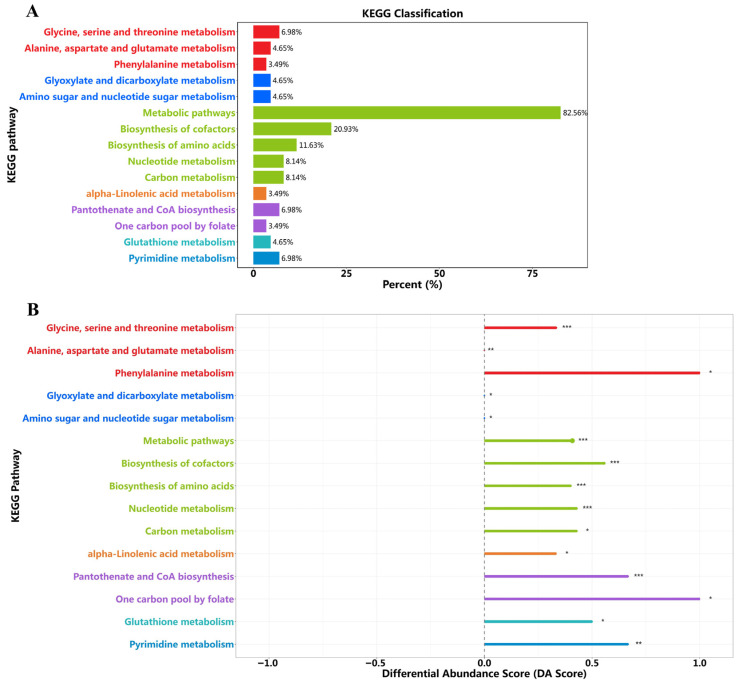
Results of the metabolic pathway analysis between C15:0 and AD groups. (**A**) Proportion of KEGG pathways of differential metabolites, (**B**) KEGG pathway differential abundance score plot. * (*p* < 0.05), ** (*p* < 0.01), *** (*p* < 0.001).

The metabolic pathways of dramatically altered metabolites were analyzed using the MetaboAnalyst (http://www.metaboanalyst.ca/) to gain insight into the metabolic mechanisms of C15:0. As shown in Figure 6A,B, this web-based metabolic pathway analysis can identify metabolic pathways with *p* < 0.05 and impact > 0.1 as potentially critical metabolic pathways. The significant metabolic pathways in *C. elegans* after treatment with C15:0 were determined in comparison to AD.

Compared with the AD model group, KEGG pathway enrichment analysis of differential metabolites in the C15:0-treated group revealed that metabolic pathways constituted 82.56% of all annotated pathways (Figure 6A), with cofactor biosynthesis (20.93%), amino acid biosynthesis (11.63%), nucleotide metabolism (8.14%), and carbon metabolism (8.14%) representing the top four enriched categories. These findings indicated that C15:0 exerts a broad, system-level modulation of the globally dysregulated metabolic network in AD, rather than isolated pathway regulation. Consistent with this, KEGG differential abundance scoring (Figure 6B) demonstrated significant upregulation across multiple core metabolic modules. Overall metabolic pathways were significantly upregulated, supporting the C15:0-mediated systemic restoration of metabolic homeostasis and underpinning its broad-spectrum neuroprotective effects. Cofactor biosynthesis was markedly enhanced, reflecting the robust recovery of coenzyme synthesis essential for TCA cycle flux and mitochondrial ATP production, which directly corroborates the mechanism by which C15:0 rescues neuronal energy deficits. KEGG analysis also revealed significant upregulation of amino acid biosynthesis-related pathways, suggesting potential restoration of amino acid metabolic homeostasis. These metabolic alterations may contribute to supplying precursors for neurotransmitter synthesis and structural proteins that support neuronal integrity and synaptic function. Nucleotide metabolism was similarly upregulated, suggesting an enhanced capacity for nucleotide salvage and DNA repair that may contribute to neuronal genomic stability and resilience against AD-associated neurodegeneration.

## 3. Materials and Methods

### 3.1. Materials and Equipment

The algal oil enriched with OCFAs was provided by Wilmar (Shanghai) Biotechnology Research & evelopment Center Co., Ltd., Shanghai, China; the DHA algal oil was provided by a local company in Anhui. Pentadecanoic acid (C15:0; ~99%, determined by capillary GC), 5-fluoro-2′-deoxyuridine (FUDR), and TERGITOL™ solution (Type NP-40, 70% in H_2_O) were all purchased from Sigma-Aldrich Co., LLC, St. Louis, MO, USA.

A self-built nematode tracking platform equipped with a 6-megapixel camera and high-resolution lens was obtained from Huazhong Agricultural University; a gas chromatograph (model GC 7820A) was purchased from Agilent Technologies, Inc., Santa Clara, CA, USA; an ultra-performance convergence chromatography-quadrupole time-of-flight mass spectrometer (UPC^2^-Q-TOF-MS) was acquired from Waters Corporation, Milford, MA, USA; and an ultra-high-performance liquid chromatography-tandem mass spectrometer (Vanquish, Orbitrap Exploris 120) was procured from Thermo Fisher Scientific, Inc., Milford, MA, USA.

### 3.2. Determination of Glyceride Composition

The lipid composition of C15:0 algal oil was analyzed via high-performance liquid chromatography (HPLC) equipped with a differential refractive index detector (RID, Agilent Technologies, Inc., Santa Clara, CA, USA) [40]. A Sepax HP-Silica column (5 μm particle size, 4.6 × 250 mm, Sepax Technologies, Inc., Newark, DE, USA) was used; the mobile phase consisted of n-hexane/isopropanol/formic acid at a volume ratio of 15:1:0.003 (*v*/*v*/*v*), with a flow rate of 1 mL/min. The column temperature was maintained at 30 °C, and the total sample elution time was 20 min. Samples were injected at a concentration of 10–20 mg/mL with an injection volume of 20 μL. Qualitative and quantitative analyses were performed using authentic standard reference materials.

### 3.3. Determination of Triglyceride Composition

Samples were analyzed using ultra-performance convergence chromatography coupled with quadrupole time-of-flight mass spectrometry (UPC^2^-Q-TOF-MS). Chromatographic conditions were adapted from the protocols described by Gao [41] and Tu et al. [42], as follows: An Acquity UPC^2^ BEH-2EP column (150 mm × 3.0 mm, 1.7 μm; Waters Corporation, Milford, MA, USA) was employed, with a column temperature of 50 °C, autosampler temperature of 20 °C, and system back pressure of 13,790 kPa. The mobile phase consisted of eluent A (supercritical CO_2_, purity ≥ 99.99%) and eluent B (acetonitrile-ethanol, 50:50, *v*/*v*), run under gradient elution conditions at a flow rate of 0.8 mL/min. The injection volume was 1 μL.

Mass Spectrometry Conditions: Electrospray ionization in positive ion mode (ESI^+^) with MS^E^ scanning was used, covering a mass-to-charge ratio (*m*/*z*) range of 100–1200 Da. Desolvation gas was nitrogen (flow rate: 700 L/h), and collision gas was argon. The ion source temperature and desolvation temperature were set to 100 °C and 450 °C, respectively. Instrument parameters included a capillary voltage of 3 kV, cone voltage of 25 V, and cone gas flow rate of 20 L/h. Collision energies were set to 6 eV (low) and 20–40 eV (high range). Leucine enkephalin (0.2 ng/μL) was used as an external standard for accurate mass locking (performed every 30 s); its *m*/*z* value in ESI^+^ mode was 556.2771. Raw data were processed using MassLynx V4.1 software (Waters Corporation, USA).

### 3.4. C. elegans Strains and Maintenance

AD model: The strains GMC101 was purchased from the *C. elegans* Genetics Center (University of Minnesota, Minneapolis, MN, USA). GMC101 (dvIs100 [unc-54p::Aβ1-42::unc-54 3′-UTR + mtl-2p::GFP]) expresses human Aβ1-42 under the control of the body wall muscle-specific unc-54 promoter, resulting in progressive motility defects and eventual paralysis following a temperature upshift to 25 °C [43]. The strain CL2122 (dvIs15 [unc-54p(vector) + mtl-2p::GFP]) was used as the corresponding control strain, in which there was no Aβ expression in the body wall muscles.

PD model: NL5901 (pkIs2386 [unc-54p::alphasynuclein::YFP + unc-119(+)]), expressing alpha-synuclein, shows slowed movement or abnormal food perception behavior at 20 °C.

HD model: AM141 (rmIs133 [unc-54p::Q40::YFP]), expressing polyglutamine protein, exhibits behavioral abnormalities such as slow movement, paralysis or convulsions at 20 °C.

Strains were cultured and maintained on nematode growth medium (NGM) agar plates seeded with *Escherichia coli* OP50 as a food source. Pregnant adult nematodes were exposed to a sodium hypochlorite and NaOH solution for egg isolation, followed by incubation in M9 buffer for 24 h to obtain synchronized L1-stage larvae. L1-stage larvae were transferred to NGM plates supplemented with 1% (*v*/*v*) of algal oil rich in OCFAs or DHA and cultured at 20 °C until reaching the L3 larvae. Then, the temperature was shifted to 25 °C to induce the expression and aggregation of Aβ. After 24 h, 0.1% (*w*/*v*) 5-fluoro-2-deoxyuridine (FUDR) was added to the NGM plates to suppress nematode reproductive capacity. After 48 h of culture at an elevated temperature, nematodes were harvested by washing with M9 buffer.

Prior to the formal experiment, a concentration gradient screening was performed using 0.5%, 1%, and 2% (*v*/*v*) of OCFAs-enriched algal oil and DHA-enriched algal oil, respectively. The selection of the optimal concentration was comprehensively evaluated based on nematode survival rate, growth status, and locomotor recovery efficacy in the AD model. Results showed that 2% (*v*/*v*) algal oil caused mild growth stress and a slight reduction in nematode survival rate, while 0.5% (*v*/*v*) only produced a weak and statistically insignificant improvement in locomotor function. In contrast, 1% (*v*/*v*) exhibited the best overall performance: no obvious cytotoxicity was observed (survival rate > 90%), nematode growth and development remained normal, and the most significant improvement in locomotor function was achieved in AD model nematodes. Accordingly, 1% (*v*/*v*) was determined as the optimal intervention concentration for both OCFAs-enriched algal oil and DHA-enriched algal oil in the present study.

The algal oil supplementation protocol was modified as described previously [44]: To improve the solubility of algal oil, the emulsifier Tergitol (type NP-40) was incorporated into NGM agar medium prior to sterilization, resulting in a final concentration of 0.001%. This modification was applied to both control plates and algal oil-treated plates. Vehicle control NGM plates contained an equivalent volume of NP-40.

### 3.5. Locomotion Analysis

Collected nematodes were transferred to unseeded 6 cm agarose plates containing M9 buffer, followed by imaging using a 6 MP monochrome camera (FLIR Integrated Imaging Solutions, Richmond, VA, Canada) and an MVL-KF1624M-25MP 16 mm lens (Hikvision, Hangzhou, China). A 30 s video recording was acquired to document the thrashing behavior. The motility was tracked by a modified software platform, the Widefield Nematode Tracking Platform (WF-NTP, Version 1.0) [39]. Between 50 and 100 nematodes were analyzed per experimental group.

### 3.6. Determination of Aβ Deposition

The expression level of human Aβ1-42 in GMC101 strain nematodes was detected by the immunofluorescence method, and the specific operation was carried out according to the method described in the literature [45]: The nematodes were fixed in 4% paraformaldehyde and treated with collagenase/β-mercaptoethanol. One drop of primary antibody (Anti-beta Amyloid 1-42) was added to each section and incubated at 4 °C overnight. Subsequently, the head sections were incubated with a secondary antibody, goat anti-mouse HRP, at room temperature for 2 h. Fluorescence images were collected using a Zeiss Carl Zeis Ism 880 confocal microscope (Carl Zeiss, Oberkochen, Germany).

### 3.7. Untargeted Metabolomics Analysis

Metabolomic profiling was performed using liquid chromatography quadrupole time-of-flight tandem mass spectrometry (LC-QTOF-MS/MS). Each biological sample contained a minimum of 2000 nematodes, with six biological replicates per experimental group. The samples were thawed on ice, and stainless steel beads were added. Homogenization was performed using a ball mill (35 Hz) for 20 s. Then, a pre-cooled 70% methanol aqueous solution was added, mixed by vortexing, and extracted at 4 °C. The supernatant was obtained after centrifugation and used for subsequent analysis. The chromatographic separation conditions were as follows:

(1) Reverse-phase chromatography: Waters ACQUITY UPLC HSS T3 C18 column (1.8 μm, 2.1 × 100 mm, Waters Corporation, Milford, MA, USA) was used; mobile phase A was ultrapure water with 0.1% formic acid, and mobile phase B was acetonitrile with 0.1% formic acid; the flow rate was 0.4 mL/min; the column temperature was 40 °C; the injection volume was 5 μL.
(2) Hydrophilic interaction chromatography (HILIC): Waters ACQUITY UPLC BEH HILIC column (1.7 μm, 2.1 × 100 mm, Waters Corporation, Milford, MA, USA) was used; mobile phase A was a mixture of 20 mM ammonium formate, 30% water, 10% methanol, and 60% acetonitrile, adjusted to pH 10.6 with ammonia water; mobile phase B was a mixture of 20 mM ammonium formate, 60% water, and 40% acetonitrile, adjusted to pH 10.6 in the same way; the flow rate was 0.4 mL/min; the column temperature was 40 °C; the injection volume was 5 μL.

Analyses were carried out on a UPLC system hyphenated with a TripleTOF 6600 quadrupole time-of-flight mass spectrometer (AB SCIEX, Framingham, MA, USA), operating in both positive and negative electrospray ionization (ESI^±^) modes for chromatographic separation, mass measurement, and spectral acquisition. Quality control (QC) was implemented on an integrated metabolomics platform, incorporating test mixtures, retention index markers, internal standards, and pooled biological QC samples. For metabolite annotation, experimental retention indices and mass spectral data were matched against reference datasets generated from authentic standards in the JiaLib metabolite database (Biotree Technologies, Shanghai, China) using the iMAP software tool v1.0 (Biotree Technologies, Shanghai, China). The differential metabolite screening criteria were VIP > 1, *p*-value < 0.05, and FC > 1.5 or < 0.65.

### 3.8. Statistical Analysis

Most experiments including lipid composition analysis, locomotor function assay, and Aβ deposition detection were performed in triplicate to ensure reproducibility. For untargeted metabolomics analysis, six biological replicates were set per group. All the experimental data were presented as mean ± standard deviation. One-way ANOVA followed by Tukey’s HSD post hoc test were performed to identify significant differences among groups using GraphPad Prism 9 (GraphPad Software Inc., San Diego, CA, USA). Significance was indicated by symbols including * (*p* < 0.05), ** (*p* < 0.01), *** (*p* < 0.001), **** (*p* < 0.0001). NS (not significant) denotes *p* > 0.05.

## 4. Conclusions

This study systematically characterized the lipid composition of OCFAs-enriched algal oil and explored its neuroprotective effects and metabolic mechanisms in neurodegenerative *C. elegans* models. OCFAs-enriched algal oil is mainly composed of triglycerides (97.3 ± 0.6%), with C15:0 and C17:0 as the core fatty acids, and C15:0/C17:0-containing TAGs accounting for more than 90% of total TAGs. This algal oil showed obvious disease-model selectivity, which significantly improved the locomotor dysfunction of AD nematodes compared to DHA algal oil. It exerted the most pronounced beneficial effect on AD nematodes, moderate improvement in PD models, and significantly impaired locomotor function with no neuroprotective efficacy in HD models. The key component C15:0 generated propionyl-CoA via β-oxidation to replenish the impaired TCA cycle and rescue neuronal energy deficits, upregulated membrane phospholipids to promote membrane repair, and reduced abnormal metabolites to restore global metabolic homeostasis. KEGG analysis demonstrated that C15:0 globally modulated core metabolic modules including amino acid, cofactor, nucleotide, and carbon metabolism in AD. In conclusion, OCFAs-enriched algal oil with C15:0 as the active ingredient is a promising nutritional intervention for AD, which alleviates AD-related motor impairment by repairing energy metabolism and restoring metabolic homeostasis, providing a theoretical basis for developing OCFA-based functional foods against AD.

## Figures and Tables

**Table 1:** The glyceride composition of algal oil enriched in OCFAs.

Type	Content/%
Triglyceride	97.3 ± 0.6
Free fatty acid	0.9 ± 0.1
Diacylglyceride	1.2 ± 0.2
Monoglyceride	0.3 ± 0.1

Each test was carried out in triplicates, and the data were expressed as means ± standard deviation.

**Table 2:** The TAG composition of algal oil enriched in OCFAs.

Retention Time (min)	[M + Na]^+^ (*m*/*z*)	ECN	Triglycerides	Contents (%)
10.46	845.7877	38	DHA-C15:0-C13:0	1.68
10.78	959.8448	35	DHA-DHA-C15:0	8.17
11.51	859.8061	39	DHA-C15:0-C14:0	2.10
12.62	873.8129	40	DHA-C15:0-C15:0	19.48
13.50	773.7766	44	C16:0-C14:0-C14:0	0.34
		44	C16:0-C15:0-C13:0	0.60
		44	C15:0-C15:0-C14:0	0.68
13.82	887.8	41	DHA-C16:0-C15:0	10.04
14.32	875.8379	42	DPA-C15:0-C15:0	4.65
14.63	787.8	45	C15:0-C15:0-C15:0	8.60
15.02	901.85	42	DHA-C17:0-C15:0	11.20
15.57	889.8588	43	DPA-C16:0-C15:0	2.58
15.91	801.8098	46	C16:0-C15:0-C15:0	4.94
		46	C17:0-C15:0-C14:0	0.65
		46	C16:0-C16:0-C14:0	1.14
16.29	915.8776	43	DHA-C17:0-C16:0	3.91
16.85	903.8758	44	DPA-C17:0-C15:0	2.91
17.18	815.8254	47	C17:0-C15:0-C15:0	3.07
		47	C16:0-C16:0-C15:0	3.80
17.59	929.8923	44	DHA-C17:0-C17:0	2.18
18.11	917.892	45	DPA-C17:0-C16:0	1.07
18.51	829.8478	48	C17:0-C16:0-C15:0	2.41
		48	C16:0-C16:0-C16:0	1.25
19.81	843.866	49	C17:0-C17:0-C15:0	1.06
		49	C17:0-C16:0-C16:0	0.85

Note: Entries with identical retention time and [M + Na]^+^ value represent co-eluting TAG isomers, which were differentiated and identified by their unique MS/MS fragment ion profiles.

**Table 3:** The top 20 differentially expressed metabolites between the C15:0 and the AD group.

Serial Number	Metabolite	VIP	*p*	FC
1	PC 39:7	2.12	0.009	4.579
2	Propionyl-CoA	2.111	0.000	7.097
3	2-Hydroxybutyric acid	2.108	0.012	2.328
4	LPG 4:0	2.102	0.025	1.661
5	Piperolactam D	2.103	0.004	2.073
6	PI 2:0_7:0	2.082	0.027	1.772
7	Neohesperidin	2.099	0.006	2.255
8	PC 39:6	2.078	0.013	1.860
9	Betulin	2.078	0.015	1.822
10	DGCC 18:5_22:6	2.046	0.007	3.781
11	Asparagine	1.889	0.009	3.012
12	Amaroswerin	2.104	0.024	0.208
13	Methyl trimethoxycinnamate	2.114	0.029	0.152
14	Eriodictyol galloylglucoside	2.049	0.016	0.162
15	MG 15:1	2.124	0.033	0.593
16	LDGTS 10:0	2.144	0.019	0.336
17	Aztreonam	2.111	0.013	0.156
18	propanoic acid	2.103	0.009	0.324
19	MGDG O-13:1_2:0	2.114	0.008	0.595
20	Adenine, N6-3-methoxypropyl	2.134	0.012	0.506

## Data Availability

The original contributions presented in this study are included in the article. Further inquiries can be directed to the corresponding authors.

## Abbreviations

The following abbreviations are used in this manuscript:

AD	Alzheimer’s disease
*C. elegans*	*Caenorhabditis elegans*
C15:0	Pentadecanoic acid
DHA	Docosahexaenoic acid
DAG	Diacylglyceride
FFA	Free fatty acid
MAG	Monoglyceride
OCFA	Odd-chain fatty acid
TAG	Triglyceride
TCA	Tricarboxylic acid
